# Evaluation of depression symptoms among caregivers of children that take therapy in the national center for children rehabilitation and treatment during COVID-19 pandemic

**DOI:** 10.1192/j.eurpsy.2021.769

**Published:** 2021-08-13

**Authors:** F. Dobi, E. Kabili, E. Malile, F. Dragoti, I. Dorre, A. Jolla

**Affiliations:** Child And Adolescent Psychiatry, National Center for Children Rehabilitation and Treatment, Tirana, Albania

**Keywords:** Depression, COVID-19, caregivers, developmental disorder

## Abstract

**Introduction:**

Raising a child with neurodevelopmental disorder is very challenging. Furthermore COVID-19 pandemic can increase stress levels especially among people that suffer from mental health disorders. On of the high risks group are children with neurodevelopmental disorders. Studies show that these difficult, challenging times have had a negative impact on most families, which have a child with neurodevelopmental disorders.

**Objectives:**

Evaluation of depression symptoms among caregivers of children that take therapy in the National Center for Children Rehabilitation and Treatment (NCCRT) during COVID-19 pandemic

**Methods:**

The study was conducted during a two-month period March-April 2020. The sample involved 110 individuals, relatives, of children that were taking educative and rehabilitation therapy in NCCRT during last year, ambulatory or inpatients. Data were collected by clinical records and phone interviews with children’s caregiver. Instrument we used were: Demographic inventory and Hamilton Anxiety Rating Scale for anxiety symptom evaluation. All data were statistically analyzed through excel.

**Results:**

Most of individual interviewed, whom are responsible for children wellbeing were their parents, 69% of them. 56% of individuals were among 31-45 years old and 92% of them were women. Depression symptoms were slightly present. We noticed that depressive symptomatology was a bit worse in caregivers in urban areas compared with ones in rural areas.

**Conclusions:**

It is necessary supporting with special attention caregivers whom have depressive symptoms. Yet has to be evaluated the connection, if it’s present, between parents with depressive symptoms and children progress, for ones that are being supported with development therapy.

